# Supplementary material for: FERN – a Java framework for stochastic simulation and evaluation of reaction networks
Source: BMC Bioinformatics. 2008 Aug 29;9:356. doi: 10.1186/1471-2105-9-356 (PMC2553347; doi:10.1186/1471-2105-9-356)
Supplement: Additional file 1 — FERN distribution, Version 1.3. This archive contains the FERN source code and binaries as well as documentation and example models in FernML and SBML. [file 1471-2105-9-356-S1.zip › fern/doc/javadoc/fern/tools/NetworkTools.html]

NetworkTools


---


|  |  |  |
| --- | --- | --- |
| **Overview** \| **Package** \| **Class** \| **Use** \| **Tree** \| **Deprecated** \| **Index** \| **Help** | |  |
| **PREV CLASS**   **NEXT CLASS** | **FRAMES**    **NO FRAMES**     **All Classes** |  |
| SUMMARY: NESTED \| FIELD \| CONSTR \| METHOD | DETAIL: FIELD \| CONSTR \| METHOD |  |


---


## fern.tools Class NetworkTools

```
java.lang.Object
  fern.tools.NetworkTools
```

---

``` public class NetworkTools extends Object ```

Contains various helper methods dealing with the `Network` interface.

**Author:**
:   Florian Erhard

---

**Constructor Summary**

`NetworkTools()`


| **Method Summary** | |
| --- | --- |
| `static boolean` | `areEqual(Network a, Network b)`             Gets whether or not the two given networks contain the same species, the same reactions and yield the same propensities when the amounts of each reactant are respectively 1, 5, 10, 100. |
| `static void` | `dumpMathTree(MathTree tree)`             Dumps the MathTree to stdout. |
| `static void` | `dumpMathTree(MathTree tree, Writer writer)`             Dumps the math tree to the given `Writer`. |
| `static void` | `dumpNetwork(Network net)`             Dumps the network to stdout. |
| `static void` | `dumpNetwork(Network net, Writer writer)`             Dumps the network to the given `Writer`. |
| `static double` | `getConstantBySettingReactantsToStoich(Network net, int r)`             Gets the reaction rate constant by setting the reactant amounts to the stoichiometric coefficient. |
| `static String[]` | `getReactionNames(Network net, int[] reaction)`             Transforms the reaction indices in the given array to the corresponding names. |
| `static String` | `getReactionNameWithAmounts(Network net, int... reactions)`             Gets a string representation of the given reactions and the amounts of each participating species in parentheses. |
| `static int[]` | `getReactionsOf(Network net, String[] speciesName)`             Gets the reaction whose products / reactants are in speciesName |
| `static int[]` | `getSpeciesIndices(Network net, String[] speciesName)`             Transforms the species names in the given array to the corresponding indices. |
| `static String[]` | `getSpeciesNames(Network net, int[] species)`             Transforms the species indices in the given array to the corresponding names. |
| `static String` | `getSpeciesNameWithAmount(Network net, int... species)`             Gets the species names together with its actual amount in parentheses |
| `static Network` | `loadNetwork(File file)`             Loads a network from file identifying the type (FernML/SBML). |
| `static void` | `useActualAmountAsInitialAmount(Network net)`             Copies the actual amount of the species in the network to its initial amount. |

**Methods inherited from class java.lang.Object**

`clone, equals, finalize, getClass, hashCode, notify, notifyAll, toString, wait, wait, wait`

**Constructor Detail**

### NetworkTools

```
public NetworkTools()
```


**Method Detail**

### getConstantBySettingReactantsToStoich

```
public static double getConstantBySettingReactantsToStoich(Network net,
                                                           int r)
```

:   Gets the reaction rate constant by setting the reactant amounts to the stoichiometric
    coefficient.

    :   **Parameters:**: `net` - network: `r` - reaction index **Returns:**: reaction rate constant

---


### areEqual

```
public static boolean areEqual(Network a,
                               Network b)
```

:   Gets whether or not the two given networks contain the same species, the same
    reactions and yield the same propensities when the amounts of each reactant are
    respectively 1, 5, 10, 100.

    :   **Parameters:**: `a` - first network: `b` - first network **Returns:**: whether or not a and b are equal

---


### loadNetwork

```
public static Network loadNetwork(File file)
                           throws IOException,
                                  JDOMException,
                                  FeatureNotSupportedException,
                                  ClassNotFoundException
```

:   Loads a network from file identifying the type (FernML/SBML).

    :   **Parameters:**: `file` - network file **Returns:**: network object **Throws:**: `JDOMException`: `IOException`: `FeatureNotSupportedException`: `ClassNotFoundException`

---


### getSpeciesNameWithAmount

```
public static String getSpeciesNameWithAmount(Network net,
                                              int... species)
```

:   Gets the species names together with its actual amount in parentheses

    :   **Parameters:**: `net` - network: `species` - species indices **Returns:**: species name with amount

---


### getReactionNameWithAmounts

```
public static String getReactionNameWithAmounts(Network net,
                                                int... reactions)
```

:   Gets a string representation of the given reactions and the amounts of each participating species in parentheses.

    :   **Parameters:**: `net` - network: `reactions` - reaction indices **Returns:**: reaction string representation with amounts of participating species

---


### getReactionNames

```
public static String[] getReactionNames(Network net,
                                        int[] reaction)
```

:   Transforms the reaction indices in the given array to the corresponding names.

    :   **Parameters:**: `net` - the network: `reaction` - reaction indices **Returns:**: names of the reactions

---


### getSpeciesNames

```
public static String[] getSpeciesNames(Network net,
                                       int[] species)
```

:   Transforms the species indices in the given array to the corresponding names.

    :   **Parameters:**: `net` - the network: `species` - species indices **Returns:**: names of the species

---


### getSpeciesIndices

```
public static int[] getSpeciesIndices(Network net,
                                      String[] speciesName)
```

:   Transforms the species names in the given array to the corresponding indices.

    :   **Parameters:**: `net` - the network: `speciesName` - species names **Returns:**: indices of the species

---


### getReactionsOf

```
public static int[] getReactionsOf(Network net,
                                   String[] speciesName)
```

:   Gets the reaction whose products / reactants are in speciesName

    :   **Parameters:**: `net` - network: `speciesName` - names of species **Returns:**: reaction indices

---


### dumpNetwork

```
public static void dumpNetwork(Network net)
```

:   Dumps the network to stdout.

    :   **Parameters:**: `net` - network

---


### dumpNetwork

```
public static void dumpNetwork(Network net,
                               Writer writer)
                        throws IOException
```

:   Dumps the network to the given `Writer`.

    :   **Parameters:**: `net` - network **Throws:**: `IOException`

---


### dumpMathTree

```
public static void dumpMathTree(MathTree tree)
```

:   Dumps the MathTree to stdout.

    :   **Parameters:**: `tree` - mathtree

---


### dumpMathTree

```
public static void dumpMathTree(MathTree tree,
                                Writer writer)
                         throws IOException
```

:   Dumps the math tree to the given `Writer`.

    :   **Parameters:**: `tree` - mathtree **Throws:**: `IOException`

---


### useActualAmountAsInitialAmount

```
public static void useActualAmountAsInitialAmount(Network net)
```

:   Copies the actual amount of the species in the network to its initial amount.

    :   **Parameters:**: `net` - network.


---


|  |  |  |
| --- | --- | --- |
| **Overview** \| **Package** \| **Class** \| **Use** \| **Tree** \| **Deprecated** \| **Index** \| **Help** | |  |
| **PREV CLASS**   **NEXT CLASS** | **FRAMES**    **NO FRAMES**     **All Classes** |  |
| SUMMARY: NESTED \| FIELD \| CONSTR \| METHOD | DETAIL: FIELD \| CONSTR \| METHOD |  |


---
